# Polycyclic aromatic hydrocarbons (PAHs) in Chinese forest soils: profile composition, spatial variations and source apportionment

**DOI:** 10.1038/s41598-017-02999-0

**Published:** 2017-06-02

**Authors:** Jabir Hussain Syed, Mehreen Iqbal, Guangcai Zhong, Athanasios Katsoyiannis, Ishwar Chandra Yadav, Jun Li, Gan Zhang

**Affiliations:** 1 State Key Laboratory of Organic Geochemistry, Guangzhou Institute of Geochemistry, Chinese Academy of Sciences, Guangzhou, 510640 China; 2 Environmental Biology and Ecotoxicology Laboratory, Department of Environmental Sciences, Faculty of Biological Sciences, Quaid-I-Azam University, Islamabad, 45320 Pakistan; 3 Norwegian Institute for Air Research (NILU) – FRAM High North Research Centre on Climate and the Environment Hjalmar Johansens gt. 14, NO - 9296, Tromsø, Norway

## Abstract

Previous studies reported that forest ecosystems can play a vital role in scavenging anthropogenic polycyclic aromatic hydrocarbons (PAHs) and act as primary reservoirs of these environmental pollutants. The present study aimed to investigate the occurrence, spatial pattern and source apportionment of PAHs across Chinese background forest soils (O- & A-horizons). The 143 soils collected from 30 mountains showed significantly (*p* < 0.05) higher levels of ∑_15_PAHs (ng g^−1^ dw) in O-horizon (222 ± 182) than A-horizon (168 ± 161). A progressive increase in the levels of lighter PAHs was observed along altitudinal gradient, however heavier PAHs did not show any variations. Carbon contents (TOC & BC) of forest soils were found weakly correlated (p < 0.01) with low molecular weight (LMW)-PAHs but showed no relation with high molecular weight (HMW)-PAHs. Source apportionment results using PMF and PCA revealed that PAHs in forest soils mainly come from local biomass burning and/or coal combustion and attributed that forest soils may become a potential sink for PAHs in the region.

## Introduction

Forests have been suggested to play an important role in global distribution and fate of persistent organic pollutants (POPs). Forests are majorly responsible in increasing the overall atmospheric deposition rate of pollutants to the terrestrial environment which results in reduction of atmospheric concentrations in forest areas while affecting deposition in other areas^1^. This will consequently cause alteration of temperature-driven geochemical circulation of POPs due to decline in long-range atmospheric transport (LRAT) and enhancement of vapor-pressure-dependent fractionation caused by the “forest filter effect”. However the decline in atmospheric concentrations is caused at the expense of soil pollution. With forest canopy serving in enhanced deposition, forest soils are also known to be reservoir of POPs and especially high-molecular organic pollutants due to enrichment of soil organic matter in these soils.

Polycyclic aromatic hydrocarbons (PAHs) are an important group of contaminants that are released into the environment primarily through the incomplete combustion of carbonaceous materials^2, 3^. Inter compartmental distribution/transfer and to travel long distances make the PAHs to be present ubiquitously^4^. Gaseous and particulate phase PAHs are mainly affected by dry deposition and precipitation^5^. PAHs travel to colder environments at higher latitudes and altitudes, are trapped by the process of cold condensation and are consequently deposited to the soil surface^6^. Soils enriched with organic matter exhibit greater accumulative tendency towards PAHs; hence, act as major environmental sink of persistent organic pollutants for the environment^6^. PAHs fractionation can occur not only because of long range transport but fractionation has been found to be occurring at local level at short range transects from direct source point of PAHs^7^.

In addition, PAHs composition might be dependent on the way of atmospheric transport and thus controlling compositional patterns of PAHs across soils of different regions. Two major sources have been generally known globally for the identification of PAHs occurrence in soils. Of those two sources, one depicts a background indicator which is mostly dominated by natural sources including biological means, forest fires and/or volcanic eruptions and might sometimes include atmospherically diverse anthropogenic emissions. The second source pattern is led by the burning of fossil fuels which are usually composed of high molecular weight PAHs (HMW-PAHs), irrespective of the different combustion situations and fuels.

In China, urbanization rate has escalated from 36.2 to 51.3% during the past 10 years which has influenced the ways of energy uses^8^, and has become one of the top contributors of PAHs globally with recorded amount of 106 Gg and 114 Gg in 2007 and 2004, respectively^9, 10^. Several studies have been conducted in China on the concentrations and distribution of PAHs in the soils of different regions^11–14^. PAHs not only contaminate the local environment of China but also tend to atmospherically transport to neighbouring countries and regions^15, 16^.

China has 133.7 million hectares of forested land comprising of different biomass like boreal in North and tropical forests in South China^17^ and is characterized by the world’s most diverse forest systems due to a very large geographical area and climate variability. China has the world’s most diverse forest systems due to a very large geographical area and climate variability, which makes it a potential site for investigation of PAHs and the effect of their distribution by sorption to black carbon (BC) and total organic carbon (TOC). There is extensive literature present on BC which acts as a strong sorptive agent for PAHs and other planar organic compounds and effects their distribution^18, 19^. But still information is lacking on PAHs concentration in the forest soils in China and their distribution effected by BC and TOC. Based on above evidences, it seems likely that forest ecosystems of China are major sites for the accumulation of PAHs. Also levels would be higher in the soils with high population and industrial activities nearby. The main objective of this study was designed (i) to study the PAHs occurrence in forest soils across China, (ii) to investigate the spatial distribution patterns (altitudinal/latitudinal), and (iii) to identify the potential sources and transport pathways.

## Results and Discussion

### Comments on occurrence

Basic descriptive statistics of individual and sum total PAHs (ng g^−1^), TOC (*%*) and BC (*ug* g^−1^) detected in A- and O-horizons of forest soils are summarized in Table 1. Details on individual concentrations of PAHs, BC and TOC has been provided in Table S1. Mean concentrations of ∑_15_PAHs were found higher in the O-horizon (222 ± 182 ng g^−1^) than in the A-horizon samples (168 ± 161 ng g^−1^). The significantly higher (*p* < 0.05) levels of individual PAHs in the O-horizon, possibly reflects the more direct exposure to atmospheric deposition of the superficial O-horizon. For most PAHs, the concentrations displayed a large variance with maximum values nearly three times higher than minimum values (Table 1). This wide range of concentrations could be result of large spatial variance and close proximity to sources^20^.

**Table 1:** Comparative description of individual and ∑_15_PAHs (ng g^−1^), TOC (*%*) and BC (*ug* g^−1^) detected in A- and O-horizons of forest soils.

Parameters	A-Horizon		O-Horizon		*p*
	Mean ± SD	Min-Max	Mean ± SD	Min-Max	
Acenaphthylene (Acey)	2.00 ± 1.44	ND-7.70	2.90 ± 2.00	0.35–15.5	0.01
Acenaphthene (Ace)	2.46 ± 1.80	ND-16.7	2.99 ± 0.90	ND-19.6	0.31
Fluorene (Flu)	6.90 ± 2.90	0.79–32.0	9.50 ± 6.00	1.60–25.5	0.02
Phenanthrene (Phe)	34.2 ± 8.40	4.10–144	50.0 ± 15.4	9.20–163	0.00
Anthracene (Ant)	3.10 ± 2.90	0.24–15.2	4.10 ± 1.00	0.26–12.4	0.06
Fluoranthene (Fla)	26.3 ± 8.00	1.40–183	46.0 ± 10.8	2.00–216	0.00
Pyrene (Pyr)	14.4 ± 6.00	0.90–71.9	23.0 ± 11.0	0.90–110	0.01
Benzo(a)anthracene (BaA)	8.30 ± 5.00	0.40–62.3	12.7 ± 5.00	0.45–64.0	0.02
Chrysene (Chry)	11.5 ± 6.00	0.50–64.3	15.0 ± 5.00	1.30–96.2	0.19
Benzo(b)fluoranthene (BbF)	13.9 ± 6.80	0.50–77.3	17.8 ± 10.0	1.60–120	0.21
Benzo(k)fluoranthene (BkF)	6.60 ± 3.00	0.40–51.0	8.60 ± 2.00	0.07–35.4	0.17
Benzo(a)pyrene (BaP)	7.40 ± 1.60	0.13–69.1	10.5 ± 5.00	0.13–49.3	0.09
Indeno(1,2,3,c,d)pyrene (Ind)	11.7 ± 1.00	0.30–76.9	12.0 ± 1.20	0.20–51.0	0.84
Benzo (g,h,i)perylene (BghiP)	5.80 ± 4.00	0.15–47.1	5.50 ± 1.60	0.12–28.1	0.82
Dibenzo(a,h)anthracene (DahA)	10.0 ± 5.00	0.20–40.5	8.70 ± 3.60	0.10–35.0	0.45
ΣPAHs	**168 ± 161**	**10–670**	**222 ± 182**	**27**.**8–804**	**0**.**02**
HMW-PAHs	**89**.**5 ± 24**.**0**	**3**.**40–409**	**114**.**4 ± 70**.**0**	**14**.**5–516**	**0**.**13**
LMW-PAHs	**50**.**1 ± 39**.**2**	**5**.**4–195**	**66**.**5 ± 48**.**3**	**7**.**4–201**.**9**	**0**.**01**
Total organic carbon (TOC)	11.4 ± 2.60	2.39–43.7	26.7 ± 11.6	2.86–44.6	<0.001
Black carbon (BC)	2.80 ± 1.00	0.06–14.0	5.90 ± 3.70	0.17–14.2	<0.001

***p*** = Probability value, significant when < 0.05 (independent sample t-test); Σ**PAH** = sum of non-alkylated total PAHs.

**L MW-PAHs** = low molecular weight PAHs.

**HMW-PAHs** = high molecular weight PAH.

**ND** = Not Detected (<detection limit).

PAHs compositional profile is given in Figure S2 and showed three- and four-rings PAHs were the dominant groups with large proportions (>50%) in both O- and A-horizons of forest soils. Such a result was expected because low molecular PAHs are likely to be emitted from the incomplete burning of organic materials^20^. It was predictable as low molecular weight PAHs are easily transported from local sources and/or longer distance via atmosphere than higher ring PAHs. Among 15 targeted PAHs, Phe and Fla were the most dominant compounds in both organic and minerals soils (Table 1), which were more evenly distributed due to rather indirect effects of the combustion sources since these compounds are mainly emitted from wood combustion^21, 22^.

Given the classification system suggested by Maliszewska-Kordybach *et al*.^23^, a soil concentration of 200–600 ng g^−1^ PAHs signifies lower contamination and a concentration beneath 200 ng g^−1^ indicates no contamination. Σ_15_PAHs concentrations in Chinese forest soils will be placed in the range of low to moderate levels. Comparing PAHs results with previous studies are difficult because of varying sampling strategies and target compounds. Additionally, PAHs have been widely studied in rural and urban soils, however data from forested regions are relatively rare. The concentration levels of PAHs in this study are much lower or in agreement to those in the forest soils from European regions i.e. UK and Norway^24^, Swiss^25^, Austria^26^ and Germany^27^ forest soils (Table S2). However, the levels of PAHs in this study are higher than those reported for Brazilian tropical forest top-soils^28^ and rural forest soils in the Pearl River Delta from Southern China^29^. A result that may be attributed to the reduced presence of anthropogenic activities in the South China rural sites compared to the studied Chinese background sites.

The 143 forest soil samples analyzed had higher TOC levels (26.7 ± 11.6%) in O-horizon than A-horizon (11.4 ± 2.60%) while BC constituted 5.90 ± 3.70 and 2.80 ± 1.00 *μ*g g^−1^ in O- and A-horizons, respectively. TOC and BC levels in O-horizon and found higher by an average factor of 2 than those measured in the A-horizon soil samples.

### Spatial variations

**Figure 1:**
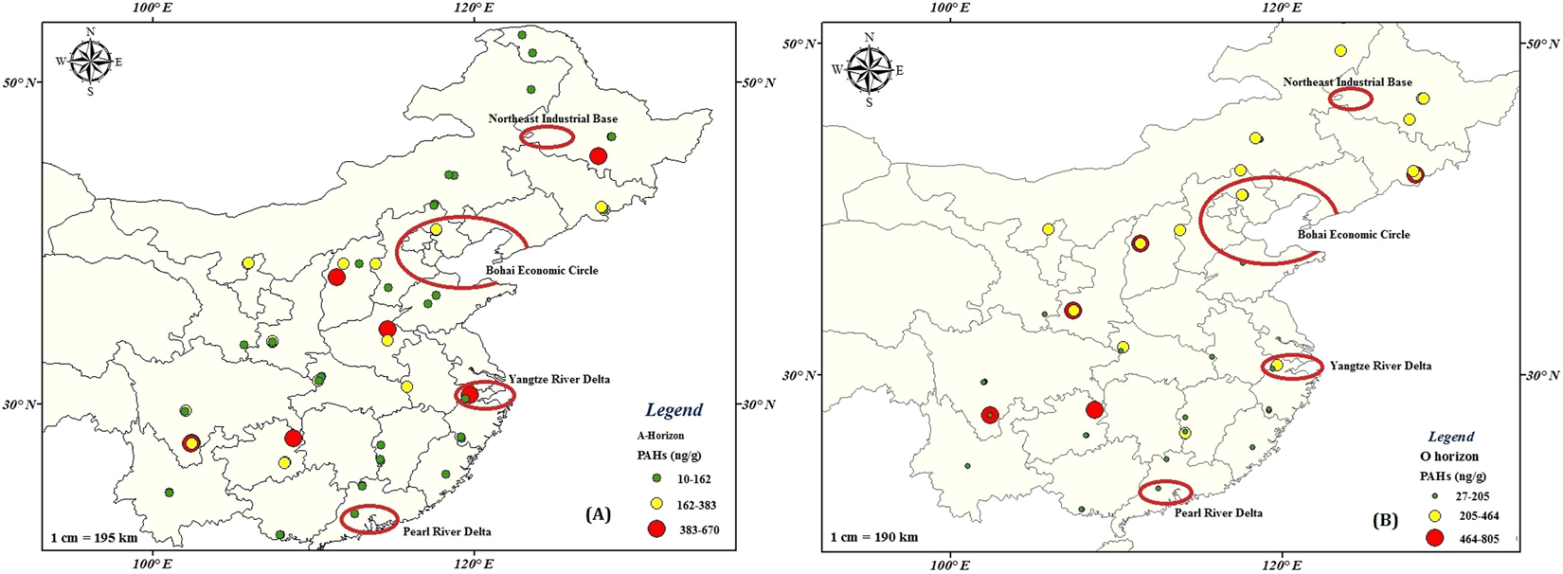
Geographical distribution of total PAHs (ng/g.dw) in soils (**A**) A-Horizon & (**B**) O-horizons across Chinese forests. (The background map was made using ArcGIS 9.3 by one co-author).

**Figure 2:**
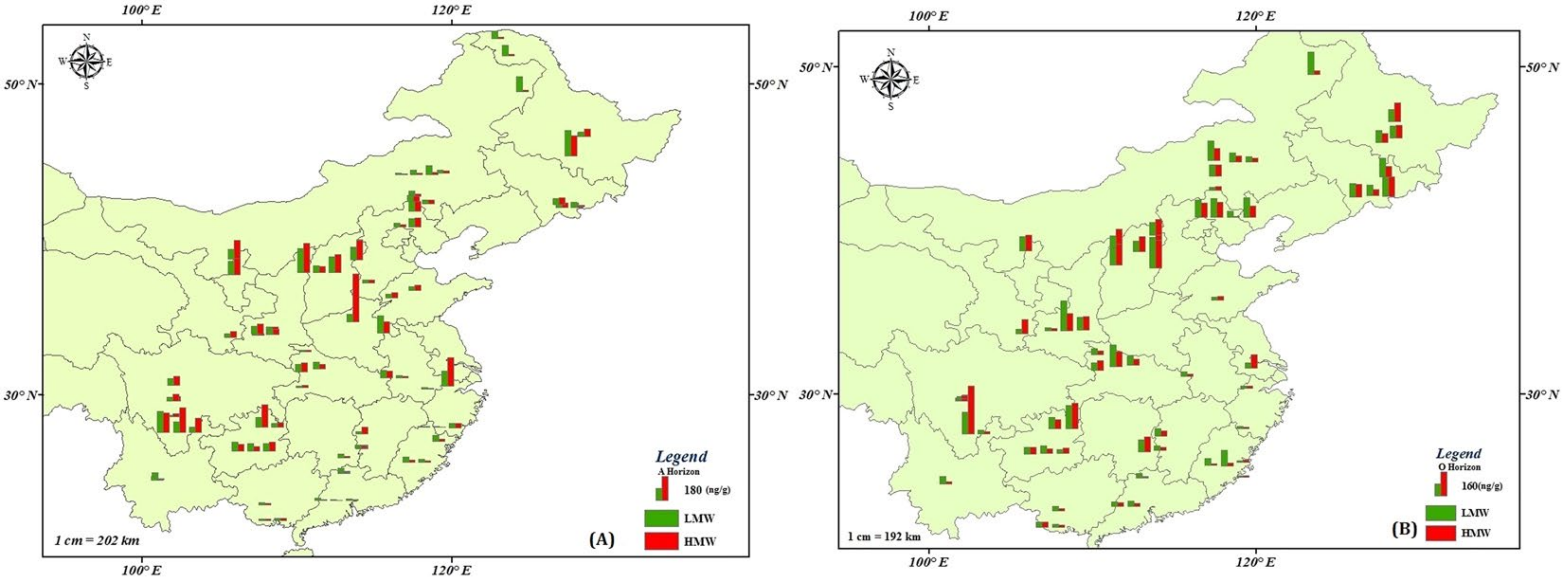
Geographical distribution of LMW- & HMW-PAHs across Chinese forest soils (**A**) A-horizon and (**B**) O-horizons. (The background map was made using ArcGIS 9.3 by one co-author).

Figure 1 is displaying the high spatial variability of ∑_15_PAHs concentrations during this survey. As discussed earlier, the ∑_15_PAHs levels have been found higher in O-horizon than A-horizon throughout the studied area which was not surprising as O-horizon of the forest soil is an organic horizon in which chronological deposition of organically bound atmospheric contaminants is largely preserved^30^. Moreover, at few sampling sites near the urban areas located in central and Western China, HMW-PAHs were found higher than LMW-PAHs (Fig. 2). Overall, the spatial pattern of total PAHs concentration showed an increasing trend from Northern to Western parts of China and decreased along the coastal areas in Southeastern China.

BC levels followed the same distribution trends of PAHs in O-horizons soils (Fig. S3). However in A-horizon, BC levels decreased significantly with the exception of few samples (i.e., 15, 26 & 57). Elevated concentrations of BC have been measured in the mineral soils collected from Anhui, Shanxi and Beijing/Tianjin areas. This trend suggests a coupling between BC and PAHs, something that has been also previously described for soils^25, 31^. TOC concentrations have been evenly distributed across the forest soils and were observed obviously higher in organic matter’s rich soils of O-layer than A-layer (Fig. S3). These findings are not surprising as PAHs in forest soils are showing the general distribution pattern and the effect of primary sources were greater than the soil organic carbon to force PAHs in soil to be associated with its contents on a nationwide scale^14, 32^.

The PAHs distribution levels were compared with those previously calculated emission inventories from China^14, 33^. Spatial distribution patterns showed that significantly higher concentrations were measured from Shanxi, Shaanxi, Sichuan, Jinlin and Heilongjiang provinces. Higher levels from these provinces indicating the impact of biomass and domestic coal combustion emissions and coal mining in the proximity of these sampling sites^33^. Emission inventories showed that relatively higher PAHs emissions come from the Southern and Eastern China, especially from the provinces around Bohai Bay, the Yangtze River Delta, and the Pearl River Delta. The PAHs emission estimates in the east part of Sichuan was also high^14, 33, 34^, while the emissions in the eastern provinces were estimated to be one times higher than that in the western provinces in China. Our findings showed that PAHs levels were very low in Southern China, except for one outlier in the forest soil samples collected from the Southeastern parts of China (i.e., Yangtze and Pearl River deltas). It showed that concentrations of PAHs in forest soils have been relatively less than in urban and agricultural soils. Compared to common soils, forest soils inevitably have lower PAHs levels owing to the effects of the forest canopy. A recent study from Pearl River Delta in Southern China confirmed that total concentrations of PAHs in forest soils decreased significantly along the urban–suburban–rural gradient, indicating the influence of anthropogenic emissions on the PAHs distribution in forest soils^29^. Forest soil is one of the main components of the multi-media atmospheres, with air, plant leaves, soil and runoff^35, 36^. It has been estimated that forest ecosystems can play vital role in scavenging anthropogenic PAHs^37^.

Recent data reviewed and published on the occurrence and primary distribution pattern of PAHs from Chinese soils^14^ revealed a positive correlation with emission inventories. Based on the results of literature over a 10 years period (2004–2013) in China, the spatial distribution of PAHs in urban and rural soil indicated an obvious geographical distribution with PAHs concentrations were found higher in Eastern China and lower in Middle and Western China^34^. However, our results do not confirm these trends and were found significantly higher in the forest soils of middle and western China indicating the local emission of these contaminants^38^. Another reason might be related to high emission intensity in the western and northern China^33^.

### Altitudinal and forest based distribution

The analysis of the altitudinal trends was based on 20 sites (86% of the entire data set), where the sampling site were sampled along the mountain slope. In these selected sites, minimum levels of 590 ng g^−1^ (mean of **∑**PAH) were observed at altitude of 521 m in the spruce bamboo forest. **∑**PAH deposition was consistently increased with increase in altitude i.e. 1913 ng g^−1^ was observed at altitude of 1700 m in the coniferous forest (mean of **∑**PAH) and 4539 ng g^−1^ (max. total PAHs) was observed at height of 3830 m in the coniferous forests.

The present result highlights a tendency for enrichment of LMW-PAHs in the forest soils at the higher altitude. HMW-PAHs did not show any trend with altitude variations. This trend was consistent across sites with different climatic conditions and confirmed some previous evidences of orographic-cold trapping collected in Chinese high mountains^39, 40^ and other regions of the world. Many factors can contribute (alone or in combination) to the observed behavior, including different sources of contamination affecting receptor locations at different altitudes, prevalence of different deposition patterns and differential scavenging of airborne contaminants over different type of vegetation along the altitudinal gradient and decreasing temperatures and increasing wet depositions at higher altitudes^6^.

Although the present dataset does not allow to investigate the influence of different drivers due to the occurrence of strong co-variance between environmental factors along the altitudinal gradient, temperature (with a maximum range of about 6° in *myt* along altitude) and wet deposition (increasing with altitude) are suggested as major influencing parameters since they both correlated with PAHs enrichment trend significantly (p < 0.05). An interesting aspect of these results is that the potential influence from local PAHs sources at lower altitudes, where human activities are concentrated, appeared not to be considerably affecting the distribution pattern of PAHs along the altitudinal gradient, rather suggesting that climate parameters are more effective drivers than proximity to primary sources.

∑PAH concentration based on different types of forest ranged from 24.81 ng g^−1^ to 4539 ng g^−1^ and these concentrations were observed in broad leaved and coniferous forests, respectively (Table S4). ∑PAH average concentration in soils of bamboo, spruce bamboo was <900 ng g^−1^ and in soils of broad leaved, birch and theropence dry forests was in between 1500–1600 ng g^−1^ while coniferous soils have average concentration of 2163 ng g^−1^. Low temperature and more acidic soils in coniferous forest (inhibiting bacterial activity) at the higher altitudes could also have affected contaminant degradation in soils favoring persistence. Furthermore, the influence of temperature is supported by the observed substance trends e.g., the temperature dependence of fugacity from soil is stronger for the heavier PAHs than lighter^41^. Lowest ∑PAH level in different types of forest was mostly <40 ng g^−1^ except for macrophanerophtes, these lower levels are in consistent with findings in rural forest soils around the world like in tropical region of Ghana (31.20 ng g^−1^)^42^, mountains of Pohang South Korea (25.6 ng g^−1^)^43^, mountains of western Canada (30 ng g^−1^)^44^, forest of subtropical mountains (33.7 ng g^−1^) and Guangzhou forest (35.86 ng g^−1^)^43^.

The source and deposition of PAHs in forest soil showed significant influence of the spatial variation on their distribution. The altitude based distribution of PAHs (individual congeners and sum of different fractions) was seen high in the middle altitude (falling between 500 and 2000 m) was highest in this study. The site distribution of PAHs revealed that almost all the PAHs species and BC and TOC were significantly higher at this altitude, than <500 and that found at >2000 for most of them. The most potent carcinogen (BaP) and sum of all 7 carcinogenic PAHs was also higher at <500 m altitudes. The phenomenal increased PAHs concentration not only reflected the long-rang transport of PAHs from the sites of origin, but also shows co-mobility of BC and PAHs (further elucidated in sections ahead) in these areas (Fig. S4).

The forest soil was generally seen with higher concentrations of PAHs compared with those found in urban areas of different cities, which is a clear indication of forest top soils being a sink of majority of fugitive PAHs from adjacent, or distant urban regions. The influx of PAHs from surroundings was largely influence by the topology of the sites serving as the sink for PAHs. The observed concentration of free and BC-sorbed-PAHs (LMW-PAHs) was significantly changed with altitudinal gradient. In fact, the deposition of PAHs was more prevalent at higher altitude, which accounted for ~13% more deposition of LMW-PAHs (Figs S4, S5). These findings also imply the prevalence of HMW-PAHs (although in lower amount) in the lower reaches of forest covers. Further, high altitude had more BC-contents (~42%) most of which were co-emergent with PAHs which depict their translocation in aggregates (i.e. BC-PAHs adsorption as discussed earlier). The regression score plot also provides evidence of ~18% LMW-PAHs were associated with BC and were abundantly translocated to higher altitudes. Having no petrogenic source in these forest, the profile of PAHs in O-horizon of provide us an evidence of urban influence on distance forest cover. In China, increasing road traffic and exhaust from petroleum combustion seem to be key factor contributing to the PAHs burden in these forests, serving as a sink for fugitive PAHs. The results also indicate possibility higher ecological risk associated with PAHs aggregation as compared to the site of their organic (urban dwellings). In supposition of forest as a major sink of PAHs deposition, and in line with the continually increasing anthropogenic release of PAHs, the concentration of PAHs in the forest sinks could only increase in future (in particular at higher altitudes).

### Relationship between precipitation pattern and PAHs distribution

The climatic conditions and natural processes such as variation in temperature, circulation of air, and changing precipitation patterns can sometimes play key (although short time) role in the distribution of contaminates in the environmental compartments. Among different weather conditions, we observed significant impact of precipitation on the occurrence of PAHs in forest soils. The log-transformed data of 7-carcinogenic-PAHs, ∑PAH, ∑LMW-PAHs, and ∑COM-PAHs were regressed against the average precipitation recorded in the sampling sites (Fig. S6). The regression scatter plot revealed that ~ 19 percent variations in the distribution of LMW-PAHs could be influenced by the precipitation pattern. It is well known phenomenon that owing to long-rang transport capabilities of PAHs (esp. that of LMW-PAHs), fractions of PAHs could be traced long distance apart from the site of their origin. The lower precipitation effect could increase the long distance translocation of LMW-PAHs, the conclusion is in compliance with the source apportionment results in this study, which also explains the abundance of PAHs in soil (see figure ratios). The low precipitation pattern was probably also influential on the distribution of ∑7-carcinogenic-PAHs, and ∑combustion origin PAHs profile observed in forest top soils.

### BC/TOC Vs PAH

Fate, transport, distribution and air-soil exchange of organic pollutants have been found to be depending on various factors in soils. However, partitioning of organic pollutants into organic matter is likely to control the sorption of the POPs to soil as reported in several studies^45–47^. Earlier studies have stated the fact that PAHs sorption can be affected by higher sorption capacities of BC than TOC in the sediments and aerosols^43, 48^. Associations between BC and PAHs are mainly due to: (a) co-release of BC and PAHs, (b) sorption of PAHs onto BC resulting due to deposition in soil, (c) PAHs gas to BC particle partitioning during atmospheric transport and (d) PAHs air to BC soil partitioning.

In the present work, the relative abilities of BC and TOC to statistically explain the distribution of PAHs in the forest soils were investigated. As given in Table S5, BC has shown a statistically positive correlation (p < 0.01) with LMW-PAHs in both O- and A-horizons (r = 0.411; r = 0.630) of forest soils (combined data from all 30 sampling sites), while TOC has only significantly correlation (p < 0.01) with lighter PAHs in O-layer (r = 0.497) soil samples. In contrast, HMW-PAHs did not show any relationship with BC in both organic and mineral soils, however, HMW-PAHs have been found weakly correlated (p < 0.05) with TOC in both O- and A- horizons (r = 0.307; r = 0.332). The weak association between HMW-PAHs and TOC depicts the abundance of the LMW-PAHs in this study and were poorly absorbed by TOC because of being volatile nature and generally they traveled short/long distance away from their site of origin^24, 31^. The close association of PAHs with BC (than TOC) indicates the proximity of sources (i.e., combustion origin)^31^, as the combustion derived PAHs are largely associated with BC (owing to their common origin in point time and space)^49^.

Briefly, correlation results indicated that PAHs reaching these Chinese background forest soils may have been emitted together with BC and may have been deposited to soils following atmospheric transport. However, as just noted, heavier PAHs have a weak association with TOC in both O- and A-horizons. This should be at least in part because BC did not show any association with heavier PAHs but it may also vary with location. Nevertheless, it can be seen that BC is less influential than organic matter in partitioning and retention of PAHs in soils. In addition, the profile of the soil horizons also revealed that the high percentage of individual and ∑PAH was associated with BC and TOC in O-horizon evidenced by the significantly higher concentration of PAHs in this zone. The high contents of BC in the O-horizon also justified the prevalence of PAHs in this compartment (in high concentration), since the distribution of BC in soil is often one of the key determinant of the abundance of PAHs^50^.

We have also done regression analysis to calculate predictive power, i.e., how much of the variation in a data set can be explained by the applied model. For assessing the roles of TOC and BC in determining the PAHs concentration in soils, regression coefficients were used (Fig. S7a,b). Overall both BC and TOC did not explain any variations for PAHs ring based distribution in Chinese forest soils. In the regression plots of BC and TOC vs. PAHs rings, a lot of scatter on the data (S7a,b) indicates that factors other than TOC or BC play a determining role in the distribution of both lighter and heavier PAHs in these back ground soils. Overall, neither BC nor TOC showed any significant impact on PAHs concentrations in the mountainous background areas which are in good agreement with previous study by Liu *et al*.^49^ from mountainous sites near Beijing. Low associations could be due to the background sampling locations which are far from the urban areas and do not receive direct PAHs/BC input from the urban area. Local coal and biomass combustion is thought to generate PAHs in the form of dispersed point sources and actually be able to dominate PAHs distribution in the vast mountain areas. Impact of local combustion sources can be seen with strong association of BC with LMW-PAHs which may explain the co-emissions of PAHs-BC along with PAHs gas-to-BC particle partitioning during short range atmospheric transport of lighter PAHs.

### Source profiling (PCA & PMF)

Multivariate pattern analysis technique and receptor model, such as Principal Component Analysis (PCA), Positive Matrix Factorization (PMF), were used to trace PAHs sources. In our study, two principal components accounted for the majority of variance (O-Horizon; 48%) of the scaled data (Fig. S8). The PCA of O-horizon confirms the findings of regression analysis as the PC-I of PAHs in the O-horizon showed a closed cluster of all LMW-PAHs and BC in close proximity. The component was dominated by Phe (0.93), Pyr (0.88) Fla (0.86) and Ant (0.85) suggested major input of mobile pyrogenic source with predominance of 3–4 ringed PAHs^29^. The results are paradoxical and the petrogenic origin of PAHs seems irrelevant to the BC sources. The association between the hydrophobic organic compounds and BC is result of their same source of origin, or their association is influenced by their affinity and other physico-chemical processes in soil and/or atmosphere. Based on this loading, it is difficult to conclude which source is dominating; albeit, the composition and relative abundance of PAHs may be altered during their transport and transfer processes^51^.

The PC-II (explaining 27% variance) clustered the BghiP (0.89), (0.86) Ind (0.87) and BaP (0.83) (Fig. S5), indicated combustion sources with dominance of HMW ring PAHs. Presence of BaP also highlighted the biomass burning as a co-dominant source in the forest soils. These finding were in accordance with the previous observations reported by Xiao *et al*.^29^ and Liu *et al*.^52^ in Chinese soils. However, many researchers linked the combustion of coal or biomass with dominantly emissions of light (2- and 3-ring) and intermediate (4-ring) molecular weight PAHs, whereas the high molecular weight PAHs (5- and 6-ring) emissions are mainly associated with traffic-related emissions^53–55^.

PCA of A-horizon (explained 71% total variance) showed almost the same cluster of PAHs, however, the BC was strongly associated with Ace, and Acy in PC-II, accounting for 20% of variance of the data. In PC-I (51%), high loadings of Flu, Pyr, Phe, BaA, DahA, BaP, BhiP, Ind, Fla and Ant suggested petroleum combustion sources of PAHs. However, presence of Ant, Pyr and Flu reflected the combustion of wood and coal. As A- horizon of soil generally represents the deposition from mobile pyrogenic and biomass combustion emissions. In PC-II, Ace, Acy and BC suggested biomass origin. Presence of Ace and Acy are also known as tracers of domestic wood combustion^2, 56^.

**Figure 3:**
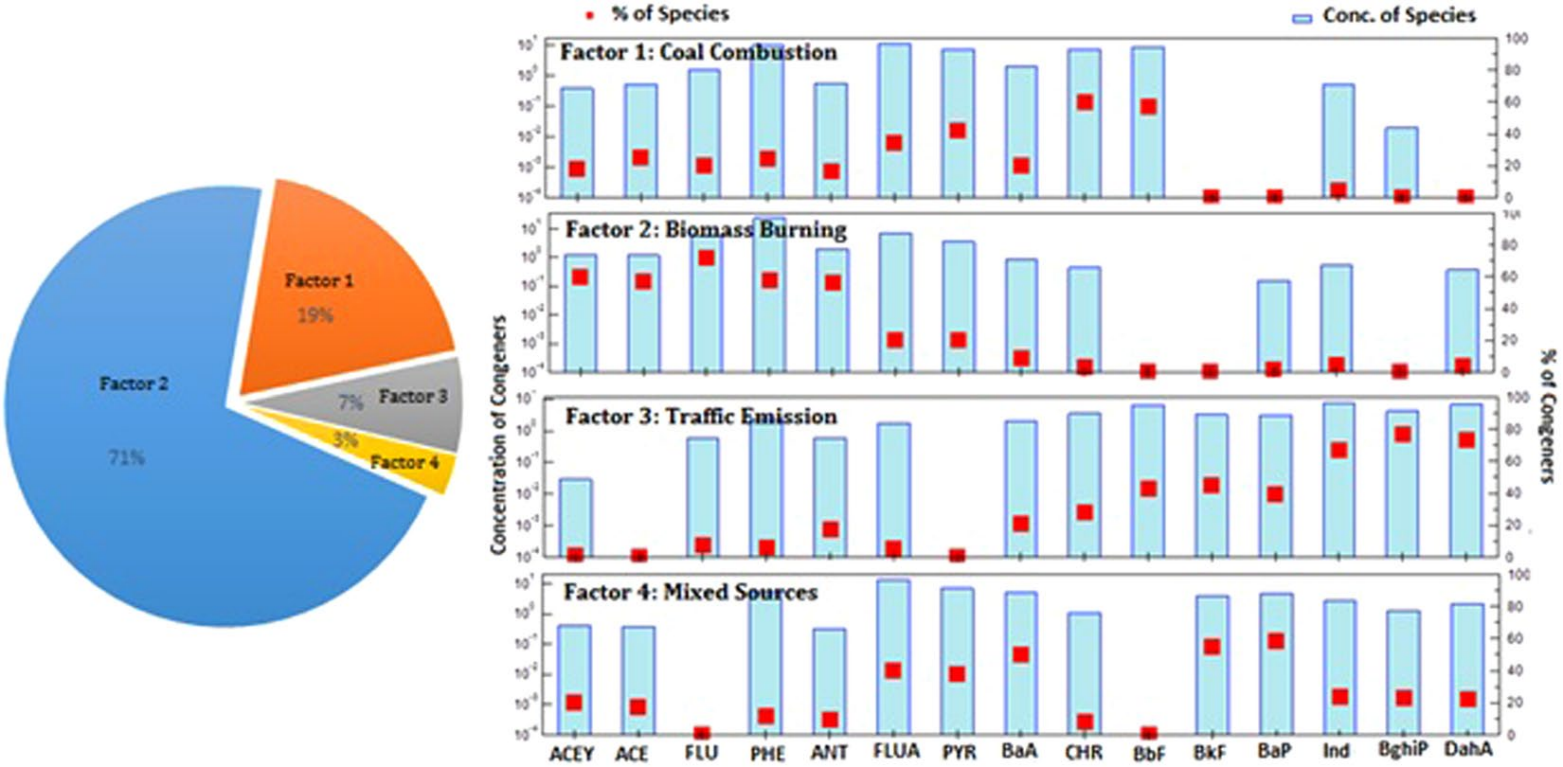
Factor pro files for PAHs sources in Chinese forest soils obtained from positive matrix factorization model.

Additionally factors (sources) (Fig. 3) and fingerprints (Fig. S9) for PAHs were identified by PMF analysis which was based on congener distribution for each source. PMF analysis tends to be better explaining the sources in comparison to PCA because PMF analysis can elucidate a better source profiling over PCA. Four factors have been identified which gave the most stable results and easily interpretable factors for the data sets. Factor 1 was greatly loaded with CHR and BbF and reasonably with PYR and FLA, contributed 19% of the total sources. Higher loadings were found for CHR and BbF which are the moderate molecular weight four rings PAHs. They have also been known as organic molecular markers of coal combustion when used in power plants, steel and iron industries and coke oven^57, 58^. Thus, factor 1 was designated to coal combustion.

Factor 2 was dominated with the high loadings of LMW-PAHs (3- & 4-ring) and represent a major proportion (71%) of the total measured PAHs (Fig. 3). It includes high loadings of ACY, ACE, FLU and ANT while PHE with middle loadings. Among these compounds, ACY and ACE are markers for domestic wood combustion^59^. This factor was highly consistent with the characteristic emission sources from burning of wood. Moreover, burning of crop straws and other vegetation have been found to be a prominent source of FLU and PHE^60^. Therefore, factor 2 was designated as biomass burning.

Factor 3 was predominantly composed of IND, BghiP and with medium loadings of BbF and BkF and contributed 7% of the total contributing sources. IND and BghiP were believed to be characteristic tracers indicative of gasoline and diesel engines emissions^61^ and lately they were recommended to characterize this source by numerous studies^62, 63^. Hence, this factor was categorized as mainly associated with traffic emissions^59^.

Factor 4 represent higher loadings of different PAHs congeners including FLA, PYR, BaA, BkF and BaP and moderate loadings of HMW-PAHs. It clearly showed mixed combustion sources i.e., both petroleum and woad/coal combustion but their contribution was the lowest (3%) among all categorized sources through PMF. These results were found in agreement with those from PCA findings and represent deposition from mobile pyrogenic and biomass combustion emission in the study area.

On the basis of our source apportionment findings, inferences can be made that Chinese forest soils might be possibly moderately PAHs contaminated with domestic biomass/coal emissions from local residential areas. However, few forest sampling sites located near the suburban/urban areas might be receiving PAHs from strong traffic emissions from the surroundings. Further, PCA results also confirmed the co-emission of PAHs and BC which might be from domestic emissions. On average, the concentration of ∑_15_PAH were exclusively high in some sites which could be considered proximal to the major emission sources and could be suitably receptacle of PAHs homologues, especially those driven by the local emission and short/long range transportation from the distant sites of origin. Our results also highlight a tendency for enrichment of PAHs in the forest soils with lower temperature and higher precipitation conditions. There are still many implications in identifying the emission sources but local sources might have the strongest influence upon PAHs fate and transport rather than other sources in Chinese forest soils.

## Materials and Methods

### Study area and sampling scheme

To collect forest soils, thirty mountainous sites were chosen across China, as details has been described previously^64^. Briefly, on the basis of altitudinal gradients and type of ecosystem, one to four sampling locations were marked at each mountainous sampling site. A total of 143 forest soils both mineral layer (A-horizon) & organic layer (O-horizon) collected during 16^th^ May, 2012 to 15^th^ March, 2013 (Fig. S1; Table S1). Environmental gradients and meteorological parameters were noted as: latitudes ranging between 21° and 53°; altitude ranging between 200 m and 3800 m; annual average temperature and total precipitation at sampling locations ranged −6 °C to 21 °C and 245 mm to 2129 mm, respectively. All the sampling locations were chosen with a minimum of tenths to several hundred kilometres (km) distance from urban agglomeration. Site wise details have been also provided in Table S1.

At each sampling site, three small trenches were excavated at the depth of 30 cm and those were located at 5 m from each other. Vegetation litter was cleared prior taking the sample without altering the surface soil. Preliminary classification of layers of soil was performed on the basis of colour and composition of the soil. Samples were collected from O-horizon and A-horizon separately from each trench using a soil auger, and then they were placed in polyethylene zip bags and immediately transported to laboratory for analysis. Mass, water content, bulk density and total organic carbon (TOC) content were determined immediately in the laboratory. Samples were freeze dried and stored at −20 °C until further analysis.

### Experimental and Chromatographic Analysis

TOC was analyzed according to the procedure explained by Chen *et al*.^65^ while for BC detection, the chemo-thermal oxidation (CTO-375) method described elsewhere^66^ was used. Details on TOC and BC analysis have been provided in the Supplementary Information (S1). For PAHs analysis, 20 g soil samples were spiked with deuterated-PAHs as recovery standards (acenapthene-D_10_, phenanthrene-D_10_, chrysene-D_12_ and perylene-D_12_) and were soxhlet extracted with dichloromethane (DCM) for 24 h, separately. Sample extract was concentrated using rotary evaporator underwent solvent exchanged to hexane with a volume of 0.5 ml. Samples were purified using alumina/silica column (8 mm i.d) closely packed with neutral alumina (3 cm, 3% deactivated), neutral silica gel (3 cm, 3% deactivated) and anhydrous sodium sulphate (1 cm). Purified fraction was eluted with 15 ml mixture of DCM and hexane (1:1 by volume) and eluted fraction was blown down under gentle stream of nitrogen (0.2 ml) to a final volume of 25 µL. 1000 ng of hexamethylebenzene (HMB) (Aldrich Chemical, Gillingham, Dorset, USA) was added as an internal standard prior to chromatographic analysis.

PAHs were analyzed by an Agilent 7890 gas chromatograph equipped with a capillary column (DB-5MS, 30 m, 0.25 mm, 0.25 μm) and a mass spectrometric detector (Mass Selective Detector (MSD), Agilent 5975). The samples (1 μL) were injected under a split less mode with a 10 min solvent delay time. High-purity helium was used as a carrier gas with a column flow rate of 1.83 mL/min. The temperature of the injector and transfer lines were 290 °C and 300 °C. The initial oven temperature was set at 60 °C for 1 min and was raised to 290 °C at a rate of 3 °C/min and held for 20 min. Sixteen PAHs were quantified that includes: Naphthalene (Nap), acenaphthene (Ace), acenaphthylene (Acy), fluorene (Flu), phenanthrene (Phe), anthracene (Ant), fluoranthene (Fla), pyrene (Pyr), benzo[a]anthracene (BaA), chrysene (Chr), benzo[b]fluoranthene (BbF), benzo[k]fluoranthene (BkF), benzo[a]pyrene (BaP), Dibenzo[a,h]anthracene (DahA), benzo[g,h,i]perylene (BghiP), and indeno[1,2,3-c,d]pyrene (Ind). However, Nap was excluded from the discussed concentration levels because of the possible evaporative losses (high detections in the blank samples) during the chemical analysis.

### Quality Control/Quality Assurance (QA/QC)

A procedural blank, a spiked blank, and a duplicate sample were run with each batch of 10 samples to assess potential sample contamination and the repeatability of the analysis. Nap was only detected regularly in the blank samples. The deuterated-PAHs recoveries for soil samples were 70% ± 11% (acenaphthene-D_10_), 82% ± 14% (phenanthrene-D_10_), 80% ± 10% (Chrysene-D_12_) and 75% ± 12% (perylene-D_12_), respectively. Detection limits for analyzed PAHs were calculated between 0.02 to 2.0 ng/g for all soil samples. All the results reported in the study were corrected for the blanks but not corrected for the surrogate recoveries. PAHs were reported as ng/g dry wt.

### Positive matrix factorization (PMF)

U.S. EPA positive matrix factorization (V5.0) was used to determine the leading sources of PAHs in Chinese forest soil. By supposing that there is no selective retention or degradation in environmental matrices between the source and deposition to environmental, PMF can be used to find out which source and how much of that source is contributing to the concentration of PAHs in different environmental matrices^67–70^. In brief, the PMF model is based on the following equations:

1 $${X}_{ij}=\sum _{k=1}^{p}{A}_{ik}{F}_{kj}+{R}_{ij}$$

where *X*_*ij*_ is the concentration of the concentration of the *j* ^th^ congener in the *i* ^th^ sample of the original data sets; *A*_*ik*_ is the contribution of the *k* ^th^ factor to the *i* ^th^ sample; *F*_*kj*_ is the fraction of the *k* ^th^ factor arising from congener j; and *R*_*ij*_ is the residual between the measured *X*_*ij*_ and the estimated *X*_*ij*_ using *p* principal components.

2 $$Q=\sum _{i=1}^{n}\sum _{j=1}^{m}{(\frac{{X}_{ij}-{\sum }_{k=1}^{p}{A}_{ik}{F}_{kj}}{{S}_{ij}})}^{2}$$

where *S*_*ij*_ is the uncertainty of the *j* ^th^ congener in the *i* ^th^ sample of the original data sets containing m congeners and n samples. *Q* is the weighted sum of squares of differences between the PMF output and the original data sets.

Before analyzing, the undetectable value (dull value) was replaced with concentration value of one half the MDL. An uncertainty of 20% for each PAHs dataset was adopted based on the results from regularly analyzing the standard reference material^71^. During the PMF analysis, a143 × 15 (143 samples with 15PAHs each) data set was introduced, the model was run for 3–7 factors and always with random seeds. For each run, the stability and reliability of the output were checked based on the Q value, residual analysis and correlation coefficients between observed and predicted concentrations. Finally, a 4-factor solution, which gave the most stable results and easily interpretable factors, was chosen for the data sets.

### Statistical Analysis

MS-Excel and SPSS (Version 21) were used for statistical analysis, principal component analysis (PCA) and regression. Further details have been given in the Supplementary Information (S2).

## Electronic supplementary material

Supporting Information

Table S1
