# Supplementary material for: Polycyclic aromatic hydrocarbons (PAHs) in Chinese forest soils: profile composition, spatial variations and source apportionment
Source: Sci Rep. 2017 Jun 2;7:2692. doi: 10.1038/s41598-017-02999-0 (PMC5457447; doi:10.1038/s41598-017-02999-0)
Supplement: Supplementary file 1 — Supporting Information [file 41598_2017_2999_MOESM1_ESM.pdf]

Supporting Information

Polycyclic aromatic hydrocarbons (PAH) in Chinese forest soils: profile composition, spatial variations and source apportionment

Jabir Hussain Syed[1], Mehreen Iqbal[2], Guangcai Zhong[1], Athanasios Katsoyiannis[3], Ishwar Chandra Yadav[1], Jun Li[1], Gan Zhang[1]*

[1]State Key Laboratory of Organic Geochemistry, Guangzhou Institute of Geochemistry 510640, Chinese Academy of Sciences, Guangzhou, China
[2]Environmental Biology and Ecotoxicology Laboratory, Department of Environmental Sciences, Faculty of Biological Sciences, Quaid-I-Azam University, Islamabad 45320, Pakistan
[3]Norwegian Institute for Air Research (NILU) – FRAM High North Research Centre on Climate and the Environment Hjalmar Johansens gt. 14 NO - 9296 Tromsø, Norway

***Corresponding Author: Gan Zhang, PhD**
Address: State Key Laboratory of Organic Geochemistry, Guangzhou Institute of Geochemistry 510640, Chinese Academy of Sciences, Guangzhou, China
Email: zhanggan@gig.ac.cn

Tel: 86 20 8529 1508
Fax: 86 20 8529 0706

Contents of this file

Text S1 to S2
Figures S1 to S9
Tables S2 to S5

S 1. Statistical methods

In order to identify independent sources tracers (individual components), we used principal component analysis (PCA). The PCA was carried out with varimax rotation (Kaiser-Normalization), and initially the components having eigen values greater than 1 (following Kaiser's rule) were extracted. The influence (and association) of BC and/or TOC on PAHs (individual and sum-components) was analyzed using regression models. All the analyses were carried out with SPSS 18.0 and MS-Excel software, while the geographical distribution of PAHs concentrations in study area was plotted using ArcGIS 9.2 software.

S2. BC and TOC analysis

Total organic carbon was analyzed according to the procedure explained by Chen et al. (2009)[1]. In brief, treatment with 10% HCL was carried out for 3g of soil sample which was freeze-dried, ground and sieved, to remove the inorganic carbon, and then samples were washed three times with deionized water, and dried overnight at 60 °C. The overall standard

deviation of measurements was better than 3% (n=3). TOC contents were determined by using the TOC-VCPN with the solid sample module (SSM- 5000A; Shimadzu, Japan).

For black carbon (BC) detection, the chemo-thermal oxidation (CTO-375) method described elsewhere[2,3] was used. Briefly, the dried soil samples (2-3 g) were exposed to thermal oxidation (375 C, 18 h) in a muffle furnace under constraint air flow. They were then digested with 1 N HCL[4]. The residual organic carbon content was determined as BC by using a TOC analyzer (SSM- 5000A; Shimadzu, Japan).

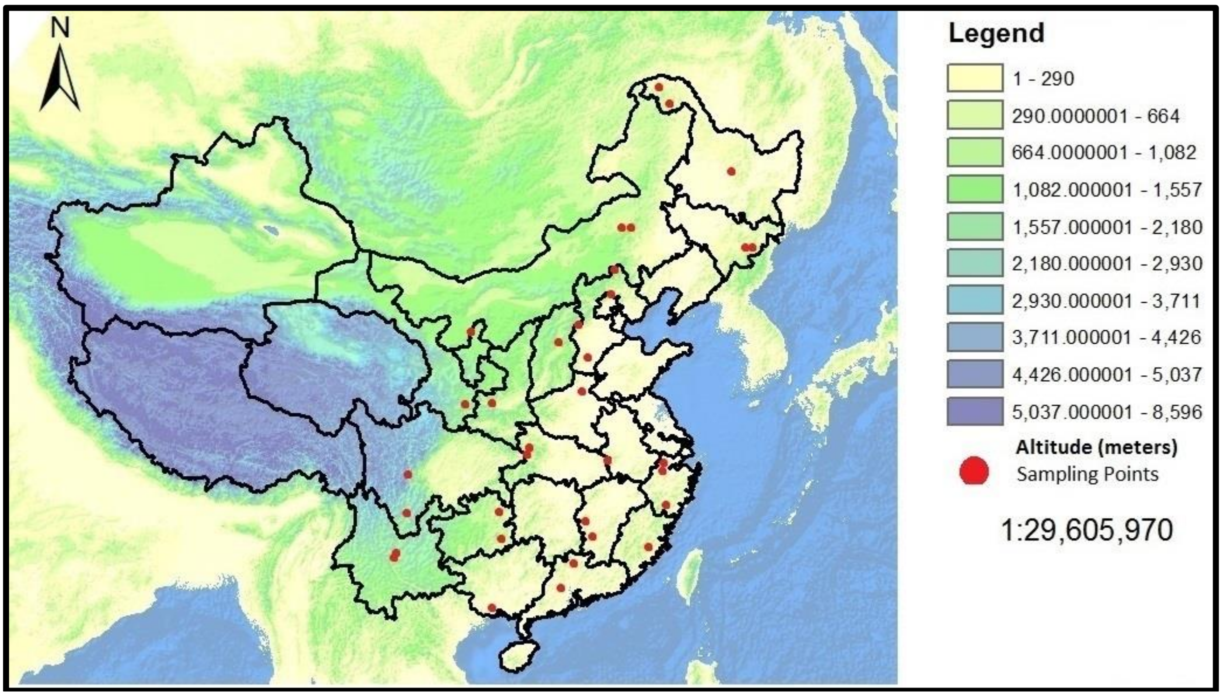

Figure S1: Altitudinal map displaying sampling sites across the forests of China. (The background map was made using ArcGIS 9.3 by one co-author)

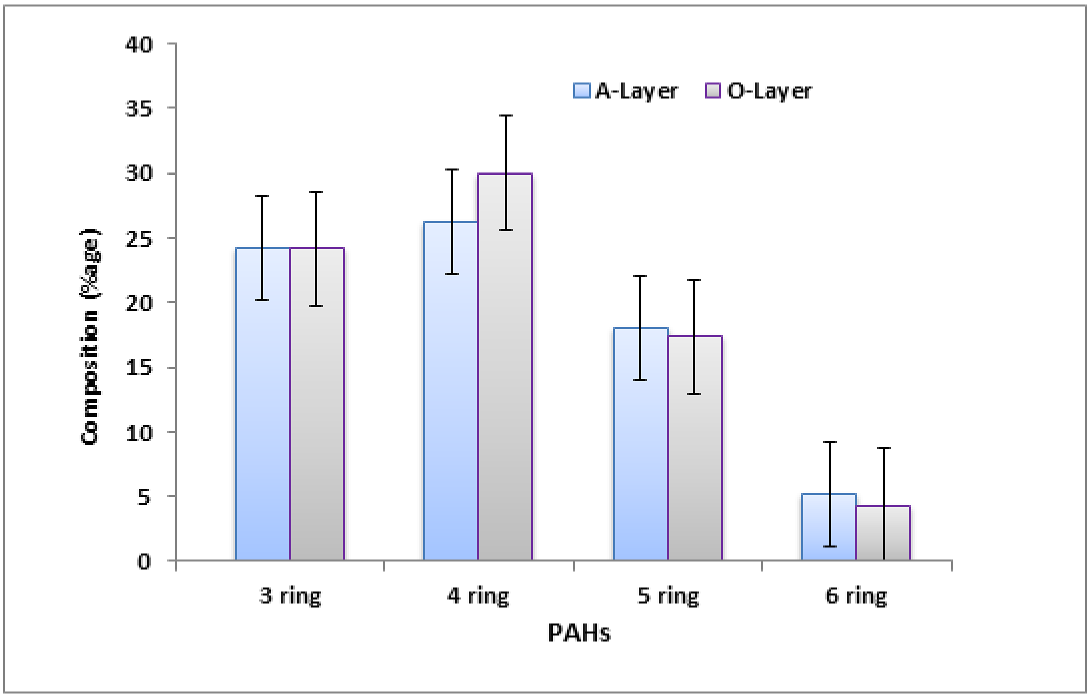

Figure S2: Composition profile of total PAHs (O- & A-horizon)

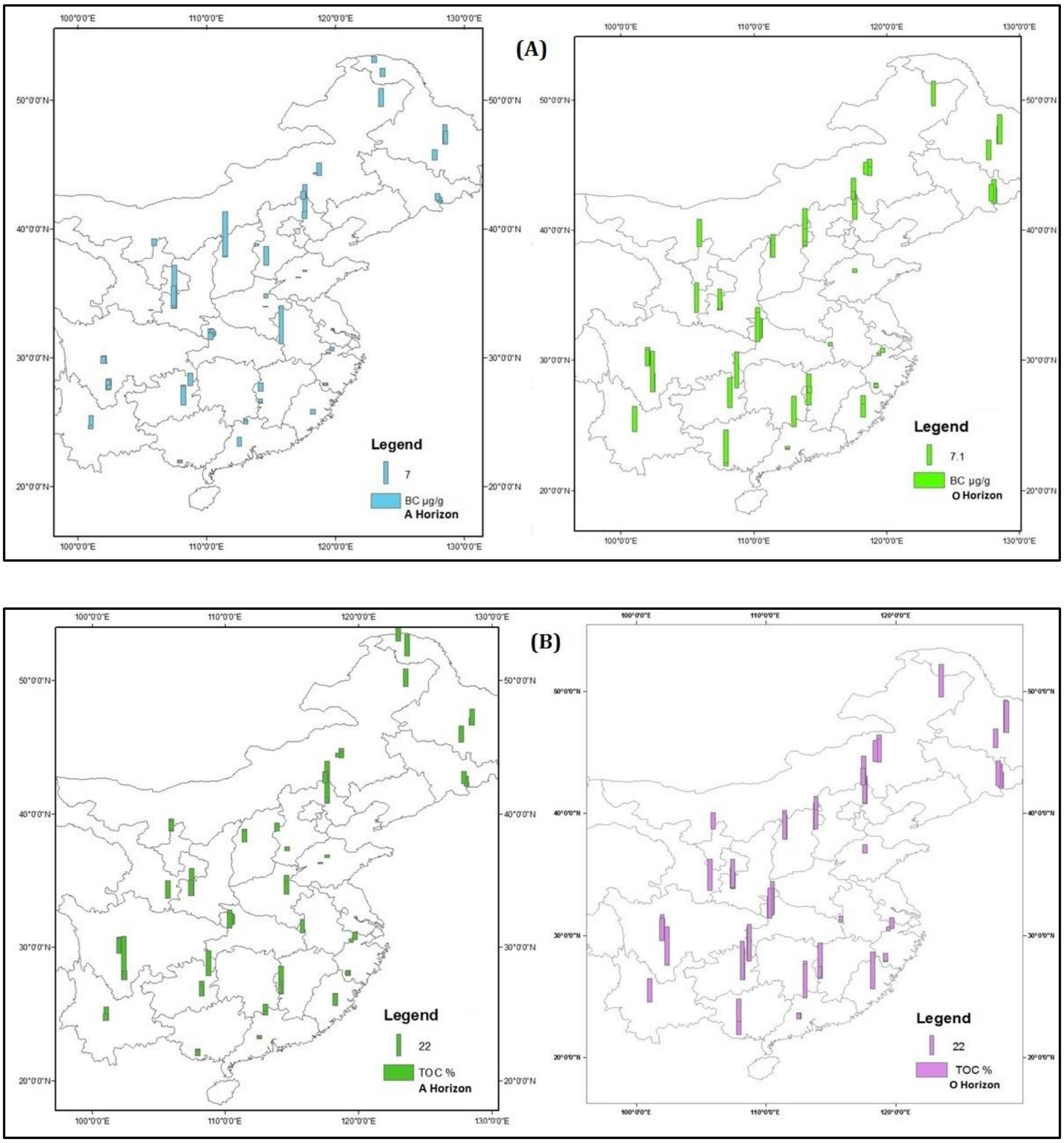

Figure S3: Spatial distribution of BC (A) and TOC (B) in the studied soil samples. (The background map was made using ArcGIS 9.3 by one co-author)

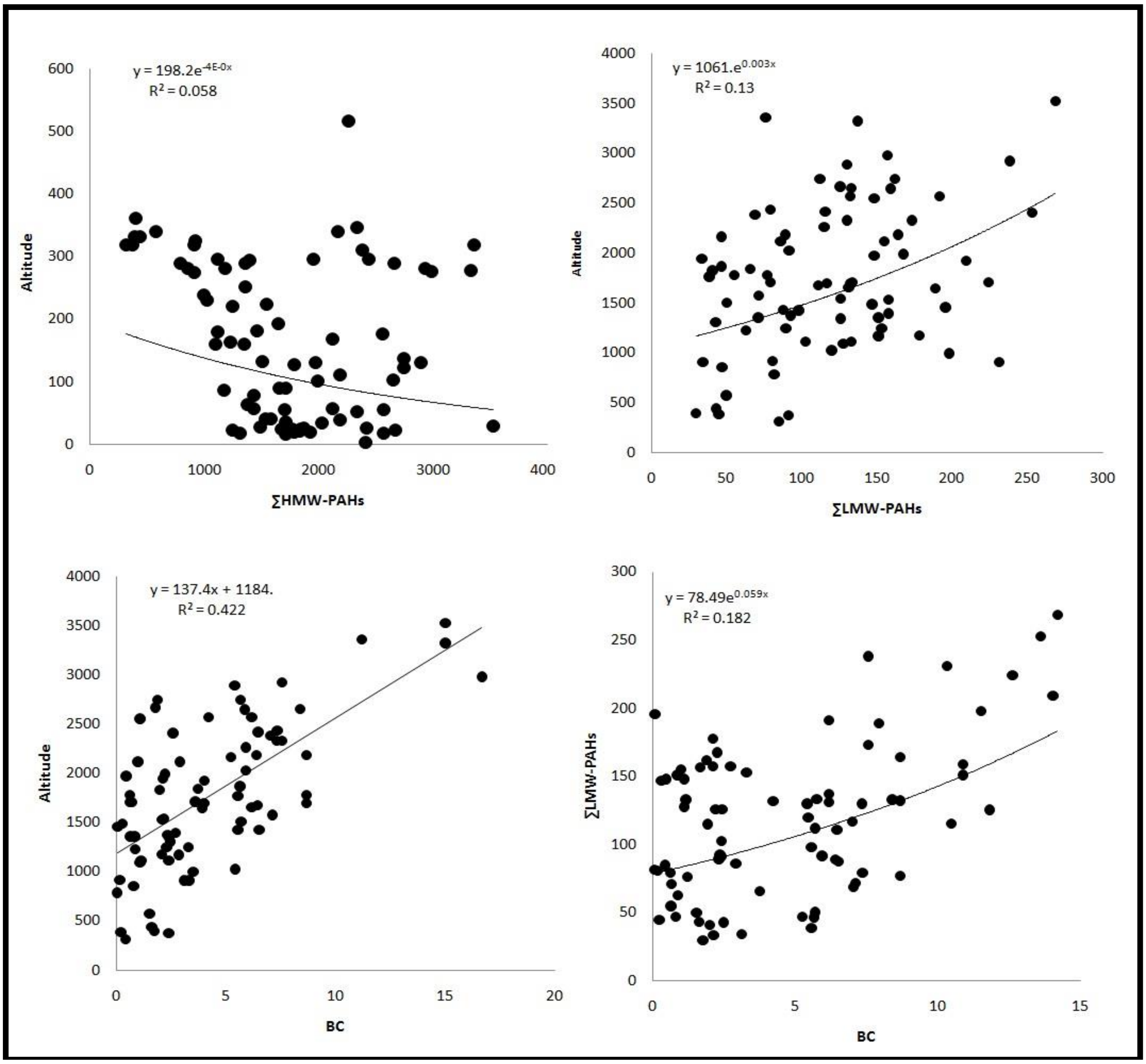

Figure S4: Topographic deposition trend of LMW vs. HMW-PAHs in Chinese forest soil

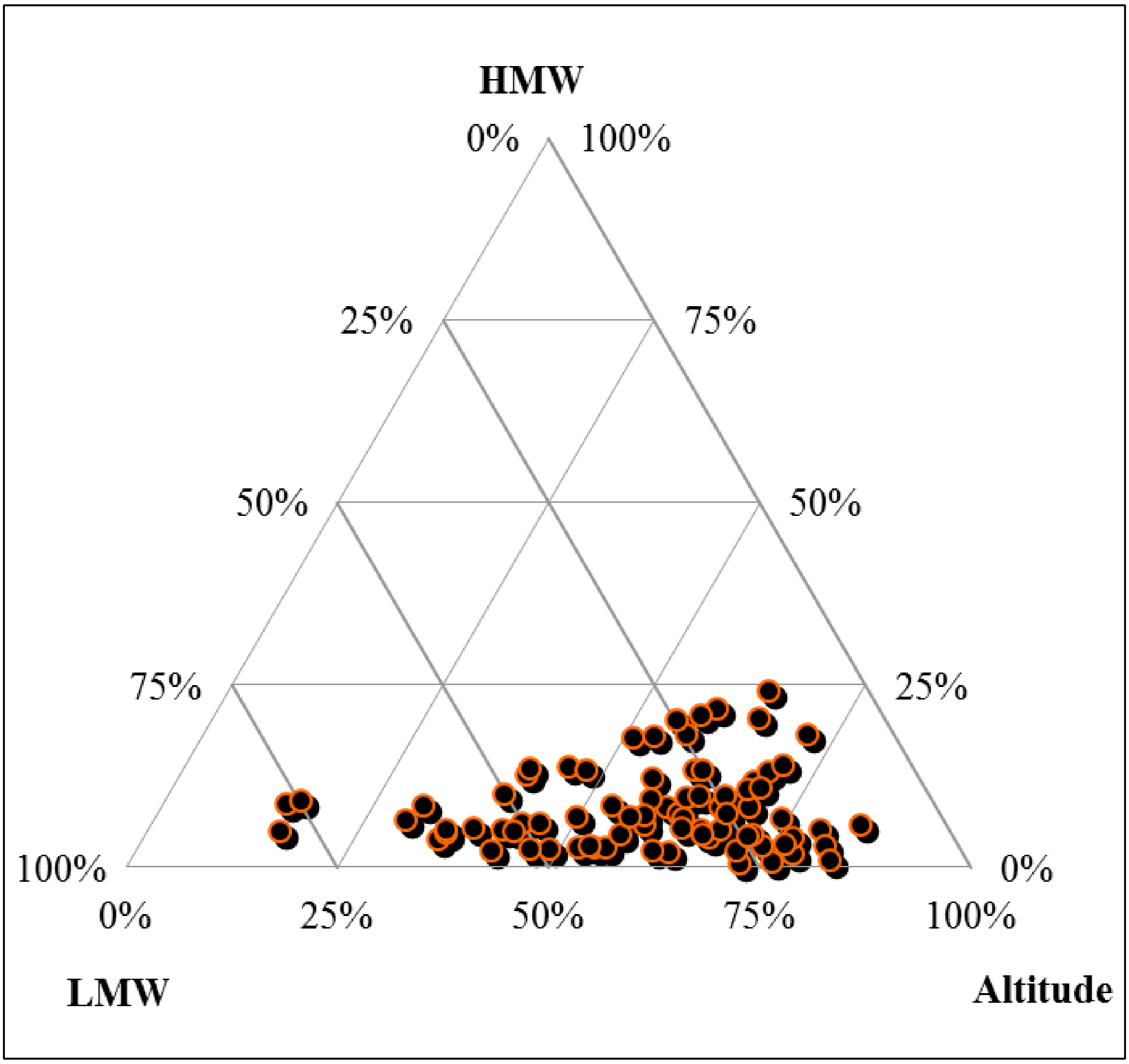

Figure S5: Triangular of Altitude distribution (in percentage) of the LMW and HMW- PAHs

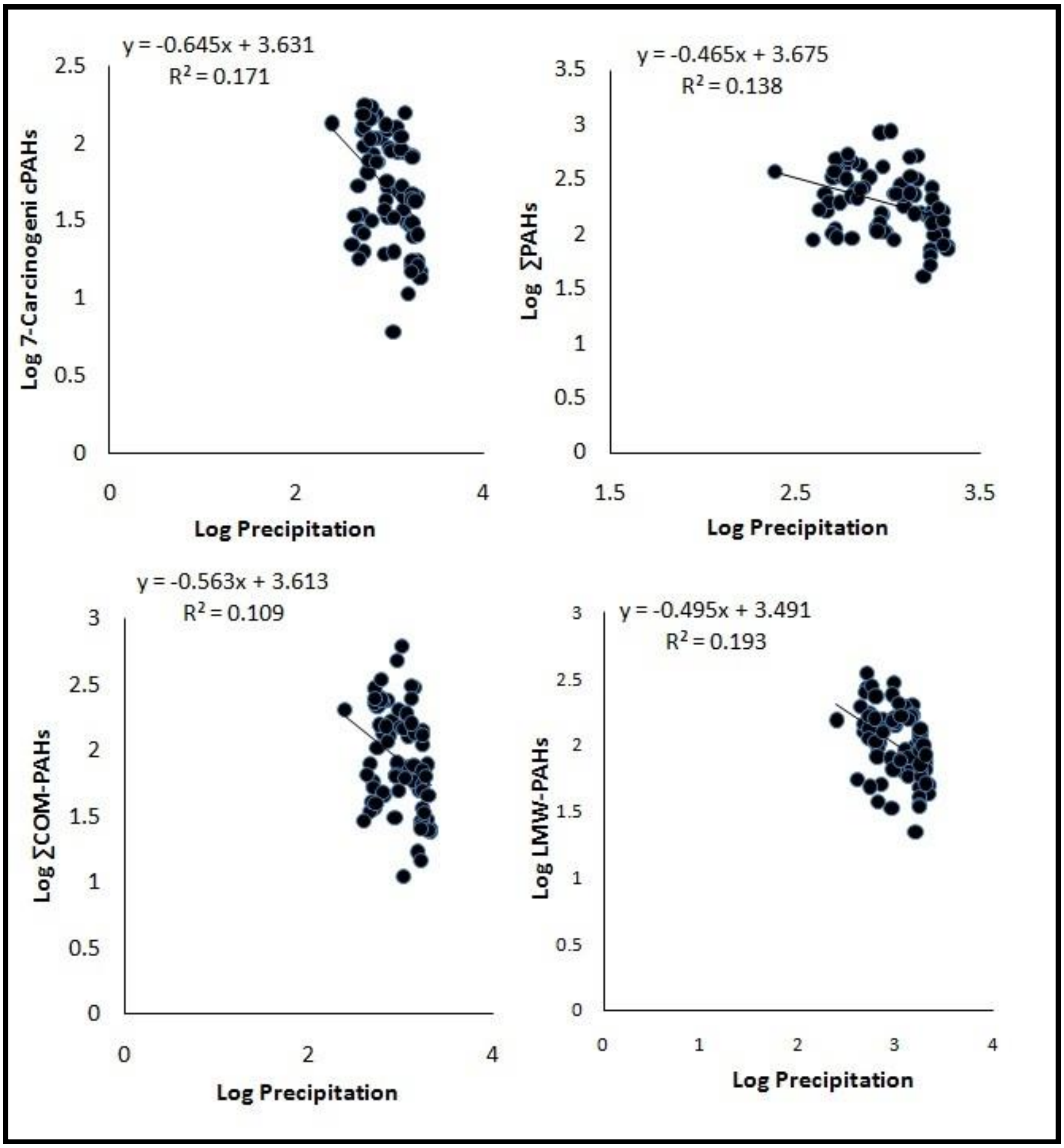

Figure S6: Influence of precipitation pattern on the distribution of PAHs

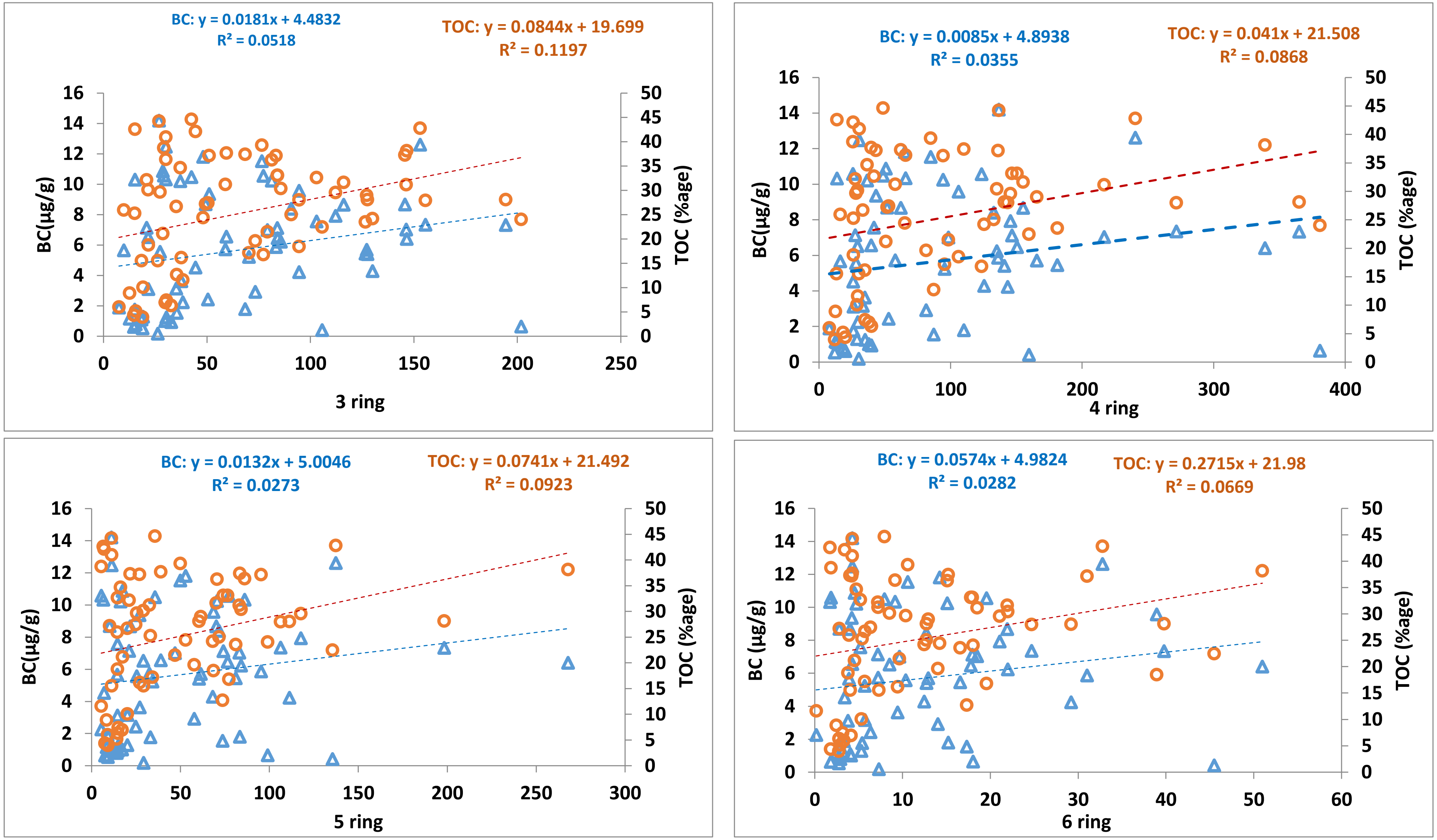

Figure S7 (a): Regression plots of TOC and BC against PAH benzene ring numbers for O- Layer soils

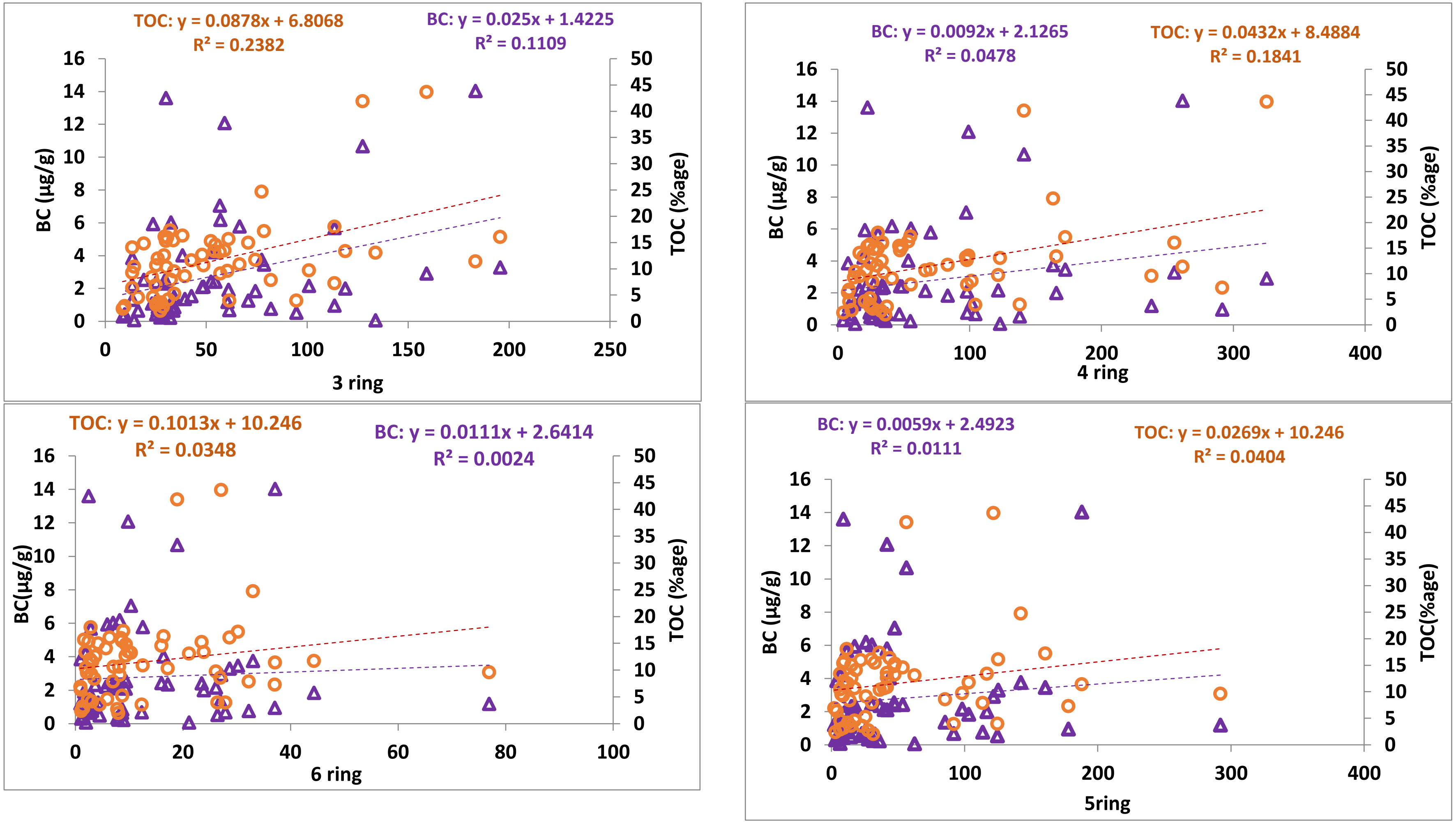

Figure S7 (b): Regression plots of TOC and BC against PAH benzene ring numbers for A- Layer soil

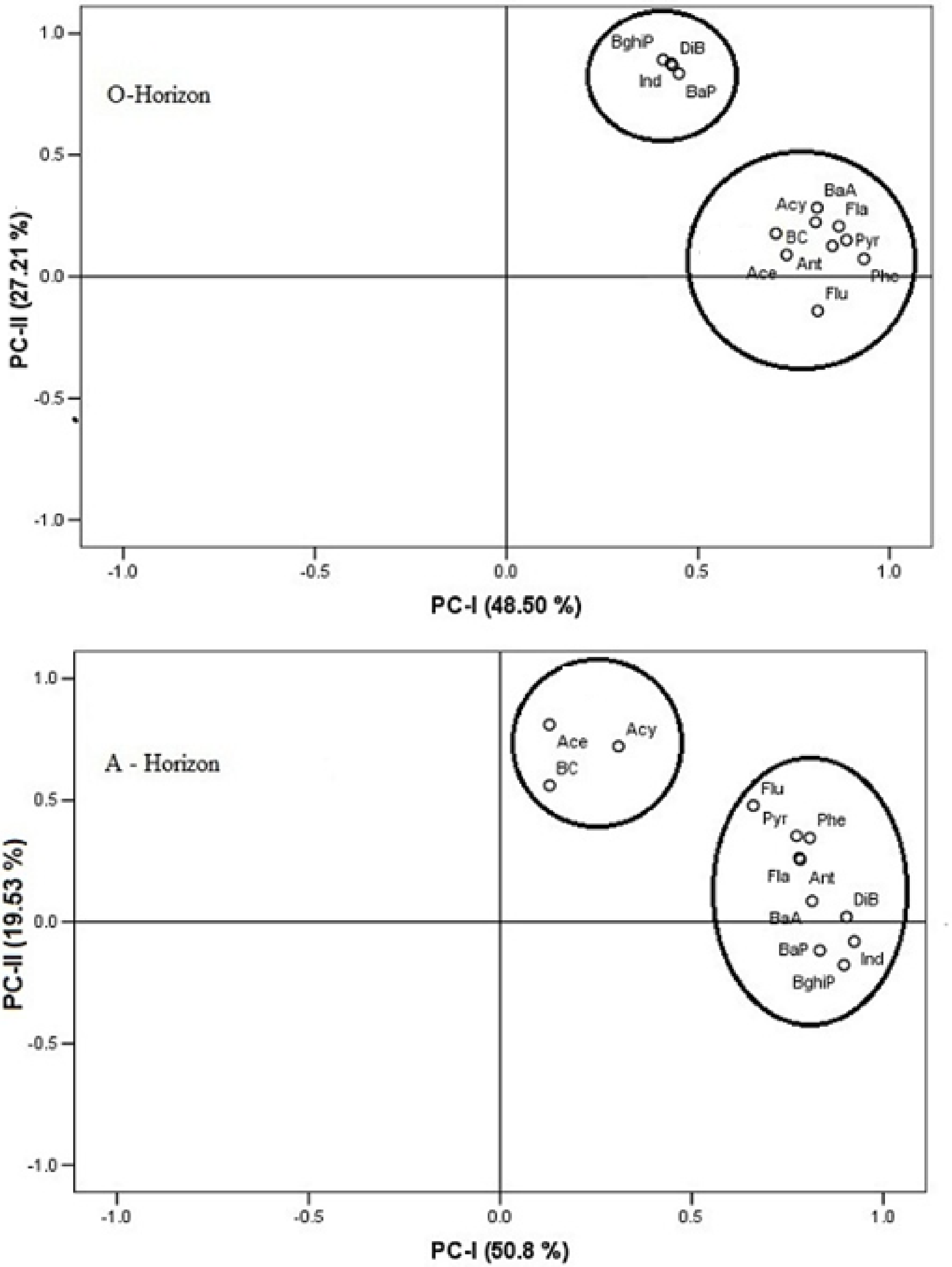

Figure S8: PCA bi-plot of PAHs congeners and BC contents in O- & A-horizon in soils of Chinese forests

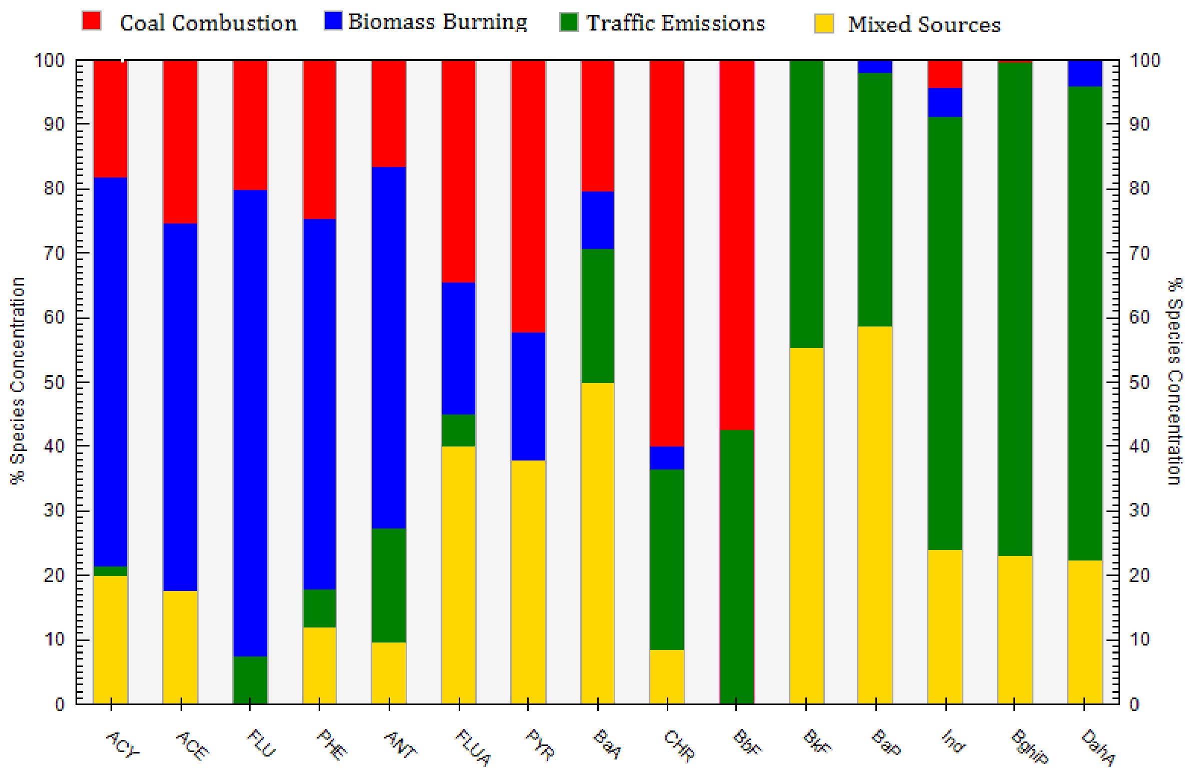

Figure S9: Fingerprints of four factor profiles on each PAHs congener in Chinese forests

Table S2: Comparison of PAH concentrations (ng g^{-1}dry weight) in the mountainous soils of different regions

Country	Sampling site	Sampling year	Total PAH	PAHs (ng g^{-1})	Reference
**UK**<br>**Norway**	Remote woodland	1998	15	580 (42–4850)<br>243 (42–750)	(Nam et al., 2008)[5]
**Austria**	Remote forest	1993	16	210[a] (68–1342)	(Weiss et al., 2000)[6]
**Switzerland**	Forest	2002	16	251 (98–578)	(Bucheli et al., 2004)[7]
**Brazil**	Tropical Forest<br>Top-soil	2001	21	32	(Krauss et al., 2005)[8]
**Germany**	Forest Soils	2013	16	105-14889	(Ainer et al., 2013)[9]
**Pearl River Delta, Southern China**	Urban, Sub-Urban, Rural-Forests	2013	16	152, 74, 26	(Xiao et al., 2014)[10]
**China**	Forest Soils<br>O-layer<br>A-layer	2013	15	222 (28-804)<br>168 (10-670)	This study

[a] median of PAHs Concentrations

Table S3: General Topographic distributions of PAHs and BC in the forest cover of China

**Elevation**	**>2000m**		**500-2000m**		**<500m**	
	**Mean±SD**	**Min-Max**	**Mean±SD**	**Min-Max**	**Mean±SD**	**Min-Max**
**Acy**	2±1	0.7-5.5	3.1±1.7	0.6-15.5	2.2±0.9	0.4-8
**Ace**	3.3±1.7	0.5-16.6	3±2.9	0.4-19.6	2.3±2.8	0.2-13.7
**Flu**	6.4[a]±1.8	1.3-21	11.7±7.5	2.1-32	5.6±1.8	0.8-276
**Phe**	38[a]±16.9	6.3-163	55[b] ±21	7.6-148	31±17.3	4.9-144
**Ant**	2.8[a]±1	0.5-9	5[b] ±3.3	0.7-15	2.6±1.4	0.6-10
**Fla**	45[a]±23	4.3-216	39[b] ±8	3.3-149	27.9±11	3.6-132
**Pyr**	19.7±150	2.6-110	22[b] ±13	1.9-77	15.2±6.8	1.8-72.5
**BaA**	9±5.4	1-43.7	13.5[b] ±5	1.1-64	8.1±5.4	0.5-62.3
**Chr**	16.7±11	0.8-91	16[b] ±12	1.4-96	8.4±3.8	0.5-46.5
**BbF**	22[c]±16	0.6-120	19[b] ±11	1.6-94	9.6±4.4	0.7-42
**BkF**	9±1.8	0.1-49	9[b] ±7.2	0.1-26	5.7±3.7	0.5-52
**BaP**	12[c]±7.1	0.2-69	10[b] ±8.4	0.1-29	6.2±2.7	0.5-62
**Ind**	15±6.8	0.9-77	14.9[b] ±7	0.2-46	7.9±4	0.9-37
**BghiP**	7.8[c]±4	0.3-47	6.9[b] ±6.2	0.1-22	3.7±1.8	0.5-21
**DahA**	11.9±4	0.6-71	11.9[b] ±8.8	0.2-49	6.3±2.3	0.7-35
**$\sum_{16}$PAHs**	276±56	87.9-865	296[b] ±163	53.9-870	189±60	24.9-721
**∑LMW-PAHs**	107[c]±221.9	33.6-340	131[b] ±58	29.9-268	90±221.7	8.2-312
**∑HMW-PAHs**	124[c]±48	7.4-516	125[b] ±94	17.9-454	71.1±276.7	7.5-375
**TOC**	20.5±6	2.9-44	21.5[b] ±5.9	3.6-45	15.2±5.2	2.4-42.6
**BC**	4.8±1.4	0.1-14	4.9[b] ±1.5	0.5-14	3.5±0.8	0.1-12.5

[a]Significantly higher in >2000m, [b] Significantly higher at altitude between 500 and 2000 m, [c]Significantly higher at >2000 than at 500

Table S4: Forest based ∑PAHs (ng/g) distribution in Chinese forest soils

	**Coniferous forest**	**Broad-leaved**	**Theropencedrymion**	**Birch**	**Mesophorbium**	**Macrophanerophytes**	**Bamboo**	**Dahurian larch**
**Min**	751	25	72	191	-	-	-	-
**Max**	4539	3601	2684	2587	-	-	-	-
**Mean**	2163	1509	1566	1600	1763	143	831	2356
**SD**	900	945	598	793	-	-	-	-

Table S5: Pearson correlations based on molecular weight PAHs versus BC and TOC

O-Layer	TOC	BC	LMW	HMW	Precipitation	Temperature
**TOC**	1					
**BC**	0.767**	1				
**LMW**	0.497**	0.411**	1			
**HMW**	0.307*	0.186	0.769**	1		
**A-Layer**	**TOC**	**BC**	**LMW**	**HMW**		
**TOC**	1					
**BC**	0.370**	1				
**LMW**	0.189	0.630**	1			
**HMW**	0.332*	0.155	0.158	1		
**Precipitation**	-0.1	-0.144	0.156	0.8	1	
**Temperature**	0.560**	-0.65	0.142	0.15	0.672**	1

** $p< 0.01$ * $p< 0.05$

References

1 Chen, L. *et al.* PBDEs in sediments of the Beijiang River, China: levels, distribution, and influence of total organic carbon. *Chemosphere* **76**, 226-231 (2009).

2 Elmquist, M., Semiletov, I., Guo, L. & Gustafsson, Ö. Pan-Arctic patterns in black carbon sources and fluvial discharges deduced from radiocarbon and PAH source apportionment markers in estuarine surface sediments. *Global Biogeochemical Cycles* **22** (2008).

3 Gustafsson, Ö. *et al.* Evaluation of a protocol for the quantification of black carbon in sediments. *Global Biogeochemical Cycles* **15**, 881-890 (2001).

4 Elmquist, M., Zencak, Z. & Gustafsson, Ö. A 700 year sediment record of black carbon and polycyclic aromatic hydrocarbons near the EMEP air monitoring station in Aspvreten, Sweden. *Environmental science & technology* **41**, 6926-6932 (2007).

5 Nam, J. J. *et al.* PAHs in background soils from Western Europe: influence of atmospheric deposition and soil organic matter. *Chemosphere* **70**, 1596-1602 (2008).

6 Weiss, P., Lorbeer, G. & Scharf, S. Regional aspects and statistical characterisation of the load with semivolatile organic compounds at remote Austrian forest sites. *Chemosphere* **40**, 1159-1171 (2000).

7 Bucheli, T. D., Blum, F., Desaules, A. & Gustafsson, Ö. Polycyclic aromatic hydrocarbons, black carbon, and molecular markers in soils of Switzerland. *Chemosphere* **56**, 1061-1076 (2004).

8 Krauss, M. *et al.* Atmospheric versus biological sources of polycyclic aromatic hydrocarbons (PAHs) in a tropical rain forest environment. *Environmental Pollution* **135**, 143-154 (2005).

9 Aichner, B., Bussian, B., Lehnik-Habrink, P. & Hein, S. Levels and spatial distribution of persistent organic pollutants in the environment: a case study of German forest soils. *Environmental science & technology* **47**, 12703-12714 (2013).

10 Xiao, Y., Tong, F., Kuang, Y. & Chen, B. Distribution and source apportionment of polycyclic aromatic hydrocarbons (PAHs) in forest soils from urban to rural Areas in the Pearl River delta of Southern China. *International journal of environmental research and public health* **11**, 2642-2656 (2014).
